# Hornification of Softwood and Hardwood Pulps Correlating with a Decrease in Accessible Surfaces

**DOI:** 10.1021/acsomega.5c03470

**Published:** 2025-06-11

**Authors:** Carl Moser, Björn Sjöstrand

**Affiliations:** † Wallenberg Wood Science Center, Department of Fibre and Polymer Technology, 7655 KTH Royal Institute of Technology, SE-100 44 Stockholm, Sweden; ‡ Chemical Engineering, Department of Engineering and Chemical Sciences, 4209 Karlstad University, 651 88 Karlstad, Sweden

## Abstract

This study aimed to investigate the relationship between hornification and the accessible surface area for cellulosic pulps in order to obtain a better understanding of the mechanisms behind hornification. Hardwood and softwood paper grades were hornified to varying degrees by sequential high-temperature drying cycles, and the degree of hornification was assessed by water retention, which was shown to decrease linearly and to correlate closely with accessible surface areas measured by xyloglucan adsorption for both hardwood and softwood pulps. The relationship between hornification ratios and accessible surfaces for xyloglucan adsorption shows different linear relationships above and below 100 °C, supporting the hypothesis of different hornification mechanisms at different temperatures shown in the previous literature. Furthermore, hornification was shown to cause a reduction in fiber width for softwood pulps and a reduction in fiber length for hardwood fibers.

## Graphical abstract

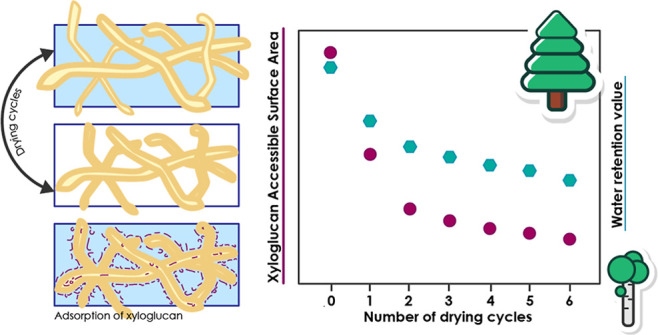

## Introduction

Drying of cellulosic materials, particularly wood pulps, involves the partially irreversible closure of small pores in the fiber wall, leading to fiber collapse and ultimately to a phenomenon known as fiber hornification.^1^ Hornification of pulps has been known for many years. It occurs when chemical bonds form between cellulose surfaces during dewatering and drying, preventing the material from reswelling in water to its original structure. In the application of wood pulp fibers for papermaking, this phenomenon results in reduced fiber wall swelling, reduced internal and external fibrillation, and reduced flexibility compared to never-dried (ND) pulp, leading to a reduced ability to form effective fiber networks with many fiber–fiber bonds, resulting in lower paper strength.^1–4^ However, hornification can also be beneficial in some applications; stiffer fibers can be an advantage in liquid packaging board, where they impart higher bending stiffness to the product.^5^ Therefore, a thorough understanding of the mechanisms of hornification and ways to design processes to enhance or inhibit it would be beneficial for the efficient use of raw materials and the number of recycling cycles.

Some researchers claim that multiple hydrogen bonds (H-bonds) are responsible for hornification, where multiple hydroxyl groups on cellulose surfaces bind to each other, making it difficult for water to break the bonds due to inaccessibility and the fact that all must be broken simultaneously.^1,6–9^ The fact that hydrogen bonds are reversible in water is one of the arguments against this hypothesis.^10,11^ Another theory is that hornification is an effect of a combination of hydrophobic interactions, van der Waals forces, and hydrogen bonding, which may explain why more disordered/amorphous cellulose is more extensively hornified than natural crystalline cellulose.^12^ It has also been suggested that hornification is related to lactone bridge formation^10^ and capillary forces.^11^ Lactone bridge formation is a form of covalent cross-linking and occurs when covalent bonds are formed between cellulose chains.^10^ Capillary forces^11^ and cocrystallization^13^ are not alternative explanations to the above hypotheses, since cocrystallization must involve noncovalent interactions such as H-bonds and van der Waals forces, and capillary forces in water are highly dependent on H-bonds—rather, they should be seen as complementary at a higher hierarchical level than the strictly molecular. An additional phenomenon that may contribute to hornification is the formation of covalent cross-links, such as hemiacetals, between hydroxyl and carbonyl groups. These linkages can form under thermal conditions and may reinforce interfibrillar contacts, potentially playing a role in the reduced reswelling and increased mechanical stability observed in hornified materials.^14^

Hornification reduced the reswelling capacity of pulps in water and was observed as a lower water retention value (WRV), which was shown to be stepwise depending on water removal,^15^ as also shown in previous literature.^1^ The dependence on drying temperatures was investigated in Sjöstrand et al.,^16^ where room temperature drying and temperatures below 100 °C gave moderate hornification, and above 100 °C, the phenomenon was accelerated. Binding appears to be temperature-dependent, and several binding mechanisms have been proposed for hornification, both driven by stepwise water removal and H-bond chain formation^15^ in combination with hydrophobic interactions^12,17^ and temperature effects.^16^

Chains of H-bonds have been previously reported by Barrios et al.,^18^ and H-bonds have a well-established ability to form chains in water.^19–21^ However, H-bonds do not explain the temperature dependence reported in several publications.^14,16,22^ As hornification is observed to occur in steps,^15^ many water–cellulose interactions are not accounted for. Drying conditions affect hornification, as shown by the decrease in fiber swelling capacity observed after successive drying cycles.^1,3,22^ Higher temperatures during drying of pulps from water have been shown to result in greater hornification, especially at drying temperatures above 100 °C.^16^ The results show at least two simultaneous mechanisms behind this effect, as high-temperature dehydration reactions could lead to covalent cross-linking of the fibers in combination with multiple hydrogen bond chains. This is supported by a yellowing of the fibers. The temperature dependence below 100 °C is more puzzling, as hydrogen bond chains are expected to decrease at higher temperatures; a possible explanation is that higher mobility on the surface cellulose chains increases the degree of bonding.^16,22^ An essential aspect of dewatering and drying involves the progressive loss of multiple molecular layers of water that are initially adhered to the cellulosic surfaces. In their native, water-swollen state, cellulose fibers are enveloped by several layers of physically and chemically bound water. These water layers act not only as a plasticizing medium but also as a physical barrier preventing close contact between fibrils, thus maintaining the open, porous microstructure of the fiber network.^9,12^ As the drying process advances, especially under conditions in which temperatures exceed 100 °C, the energy input leads to the evaporation of both free and bound water. Crucially, the removal of the water layers causes the cellulosic fibrils to draw closer together within and between fibers. Once only a monomolecular or submonomolecular layer of water remains—or none at all—the capillary forces and direct hydrogen bonding between adjacent surfaces become dominant. This results in a partial collapse of the fiber wall and the formation of irreversible interfibrillar bonds, a hallmark of hornification.^9,16^ The phenomenon is particularly pronounced during thermal drying processes, where elevated temperatures accelerate water desorption and enhance fiber-to-fiber contact through thermally activated motion.^16,22^ Thus, hornification can be understood not only as a chemical phenomenon of new hydrogen bonding but also as a physical consequence of dewatering on the nanoscale. The critical role of thermal energy in overcoming the energy barriers to water desorption, particularly above the boiling point of water, emphasizes the importance of process conditions in preserving or modifying the rehydration characteristics of cellulose-based materials.^1,9,12,16^

The most widely used method to characterize and quantify hornification is WRV, which measures the amount of water after centrifugation.^10,23^ This method has remained popular for almost a century due to its simplicity and robustness.^24^ However, there is an argument for using the fiber saturation point (FSP), a solute exclusion technique, to measure hornification, which only considers water in pores.^1,25^ Another possible technique to follow the hornification process is to use differential scanning calorimetry (DSC) to quantify the distribution and size of pores within the fibers. This would allow close monitoring of pore size distribution throughout a sequential drying series.^26,27^

Accessible surfaces, including pores, or surface area, provide a direct way to measure hornification.^28^ These techniques rely on molecules attaching to the cellulose surface, and the area they detect varies, depending on the size of the molecule. The most commonly used method is gas adsorption, but this technique requires the substrate to be completely dry. Some wet techniques are available, such as the adsorption of colored substances such as Congo red or methylene blue, but these require high salt concentrations for adsorption to occur,^29^ which can lead to a collapse of the fiber structure. Xyloglucan (XG), a hemicellulose, has a strong affinity for cellulose and can be adsorbed from aqueous solutions. Xyloglucan adsorption onto cellulose is an entropy-driven process, primarily facilitated by the release of water molecules from both the hydrated cellulose surfaces and the XG molecules themselves.^30^ Xyloglucan can be detected through complexation with iodine, which offers a simple and effective qualitative or quantitative detection technique.^31^ It has been shown to be an alternative method for the determination of cellulose surfaces in wet samples.^32,33^

In this article, xyloglucan adsorption is used in conjunction with WRV to monitor the effect of drying cycles and, consequently, hornification. Fiber dimensions were also measured, both qualitatively and quantitatively, for different degrees of hornified pulps. The aim of this work is 2-fold: (i) to test a new way of measuring hornification and (ii) to improve the understanding of the mechanisms behind this phenomenon. By implementing new measurement techniques, the work aims to capture these changes more accurately and to elucidate the underlying processes that contribute to fiber hornification in pulp materials.

## Materials and Methods

### Pulp Fibers

Never-dried, fully bleached chemical kraft pulp fibers from birch (Betula pendula/pubescens) and a mixture of Norway spruce (Picea abies) and Scots pine (Pinus sylvestris) were supplied by Gruvön Mill, Billerud AB (Grums, Sweden) with pulp consistencies of 4.4% and 7.2%, respectively, hereinafter referred to as hardwood and softwood pulps. The softwood pulp has a brightness of 89% (ISO 2470), a viscosity of 760 dm^3^/kg (ISO 5351), and a molecular weight of 222 kDa and a DP of 1200 (calculated from the Mark–Houwink–Sakurada equation using *a* = 0.9 and *k* = 0.0117 cm^3^ g^–1^),^34^ and the hardwood pulp has a brightness of 88% (ISO 2470), an intrinsic viscosity of 950 dm^3^/kg (ISO 5351), and a molecular weight of 285 kDa and a DP of 1760 (calculated from the Mark–Houwink–Sakurada equation using *a* = 0.9 and *k* = 0.0117 cm^3^ g^–1^).^34^

### Drying Procedure

The pulps were dried at 125 °C in a laboratory oven, in one–five drying cycles with disintegration and rewetting between each drying cycle. The drying temperature of 125 °C was selected because hornification is known to intensify at temperatures above 100 °C, while previous studies have shown that no significant yellowing or thermal degradation occurs at 125 °C.^16^ Two reference pulps, one never dried and one dried once at room temperature, were included for comparison. The drying process began with manual squeezing of free water to reduce the moisture content to an approximate pulp consistency of 20–30%. The pulps were then spread on blotting paper and placed in an oven set at 125 °C. A reference sample, intended for drying at 20 °C, was left at room temperature in a fume cupboard at approximately 20 °C. During the drying phase, pulp temperatures were monitored using a Mitec AT40 data logger to ensure consistent and stable conditions. All samples remained in the oven or fume cupboard until completely dry, which took a minimum of 24 h. Samples requiring additional drying cycles were rewetted at room temperature by disintegrating for 30,000 revolutions in an L&W Pulp Disintegrator (App 03), followed by squeezing and redistribution onto fresh blotting paper.

### Water Retention Value (WRV)

The water retention value (WRV) was measured before and after each drying cycle according to ISO 23714 (2014). Table 1 provides an overview of the drying temperatures, cycles, and corresponding sample names. WRV ranges were calculated to show the variation in the manuscript, defined as half the distance between the maximum and minimum values, and added as error bars around the reported means.

**Table 1:** Overview of the Drying Procedure of Softwood Kraft Pulps

sample	number of drying cycles	drying temperature (°C)
SW ND	0	
SW 0	1	20
SW I	1	125
SW II	2	125
SW III	3	125
SW IV	4	125
SW V	5	125
HW ND	0	
HW 0	1	20
HW I	1	125
HW II	2	125
HW III	3	125
HW IV	4	125
HW V	5	125

### Xyloglucan Adsorption

Xyloglucan (XG) was purified from deoiled tamarind kernel (D.N. Palani, Mumbai, India) powder by first being dissolved at 70 °C with slow addition to prevent agglomeration. Liquid ammonia (NH_3_) (Acros Organics) was introduced to inactivate native enzymes and subsequently removed by evaporation. The solution was then centrifuged to eliminate particles, and residual enzymes were removed using a Q-Sepharose. XG was concentrated by evaporation and dried via freeze-drying.

For degradation, XG was treated with Trichoderma reesei endoglucanase (Merck Millipore), an enzyme that selectively cleaves internal glycosidic bonds. The resulting polymers were analyzed using GPC/SEC (DMSO-styrene column, pullulan calibration), yielding a molecular weight of 15 kDa (^35^Brumer et al. 2004) and *R*_h_ = 234 ± 80 nm (measured at 3 g/L in a Malvern Zetasizer Pro).

Adsorption was performed on duplicates of 300 mg fibers in 50 mL of xyloglucan solution (3 g/L). The fiber–xyloglucan mixture was shaken on a shaking table for 17 h at room temperature (22 °C) and then allowed to sediment for 2 h. Aliquots of the supernatant were diluted 10 times, and duplicates of 200 μL were placed in Eppendorf tubes. One milliliter of iodine solution was added to each Eppendorf tube (0.5% iodine (99.5% sublimated, Acros) and 1% potassium iodide (99%, Acros) in deionized water diluted with 20% sulfuric acid at a 1:5 ratio of iodine to sulfuric acid). The iodine–XG mixture was left in the dark for 30 min to allow for complexation.

An XG concentration series was prepared using XG concentrations of 1, 0.5, 0.25, 0.125, and 0.065 g/L. Triplicates of 200 μL were taken from each concentration and used for calibration.

Absorbance was measured on duplicate samples at λ = 660 nm in a spectrophotometer (UV-2550 UV–vis spectrophotometer, Shimadzu, JP).

### Fiber Dimensions and Appearances

All 14 pulp samples from the study were quantitatively characterized using a Valmet Fiber Image Analyzer (Valmet FS5, Valmet AB, Karlstad, Sweden), and the fiber counts from the FS5 measurements ranged from 13,000 to 20,000 fibers for each sample. The fiber length distributions from the FS5 were divided into intervals of 0.02 mm, with each interval representing a specific range of fiber lengths. For each interval, the proportion of the total observations was recorded. The mean fiber length was determined by calculating the weighted average of the midpoints of the intervals, with weights equal to the proportion of observations in each interval. No observations were omitted, i.e., the fine fraction was included. The standard deviation was calculated from the weighted deviations of the midpoints from the mean, providing a measure of the variability in the fiber length distribution. For the fiber widths, the raw distribution data were missing, so we estimated the standard deviation to be the same percentage deviation from the mean as for the length distributions. A 95% confidence interval was constructed from the calculated standard deviations of the length distributions and the estimated standard deviations of the mean widths, based on the lowest number of observations from the FS5 measurements, 13000 images.

The undried and five-cycle dried samples (SW ND, HW ND, SW V, and HW V) were also examined qualitatively by light microscopy to observe any changes in appearance (Olympus BX51, Tokyo, Japan).

## Results and Discussion

For all drying cycles, the temperature logger showed stable drying temperatures for both room temperature and 125 °C. Note that the mean temperatures for the hardwood and softwood samples are slightly different at 126 and 124 °C, respectively. The WRV results for the different drying cycles are shown in Figure 1. There is a clear trend that the never-dried pulp has the highest WRV and that each drying cycle reduces the reswelling capacity of the pulp materials.^1^ However, in the present results (Figure 1), the WRV appears to decrease linearly rather than exponentially.^1^ The first drying cycle gives the largest decrease in WRV, and the temperature of 20 °C versus 125 °C also gives a significant difference, followed by a linear decrease in WRV. The largest drop in WRV from the first drying cycle is explained by the fact that the fibers are not able to reswell to their original state after first having been rewetted, an effect that will diminish for each cycle. This is probably due to cellulose–water interactions as it is not observed when drying from other solvents than water, as shown in two previous publications.^15,17^ Although almost in agreement with previous literature,^1,16^ the WRV is not exactly the same in absolute values, even for the very similar pulp types included in Sjöstrand et al.^16^ For both hardwood and softwood, the pulp dried five times (V) was the most hornified pulp with the lowest WRV, and the pulp dried once at room temperature (0) was the least hornified, except for the never-dried (ND) pulps.

**Figure 1:**
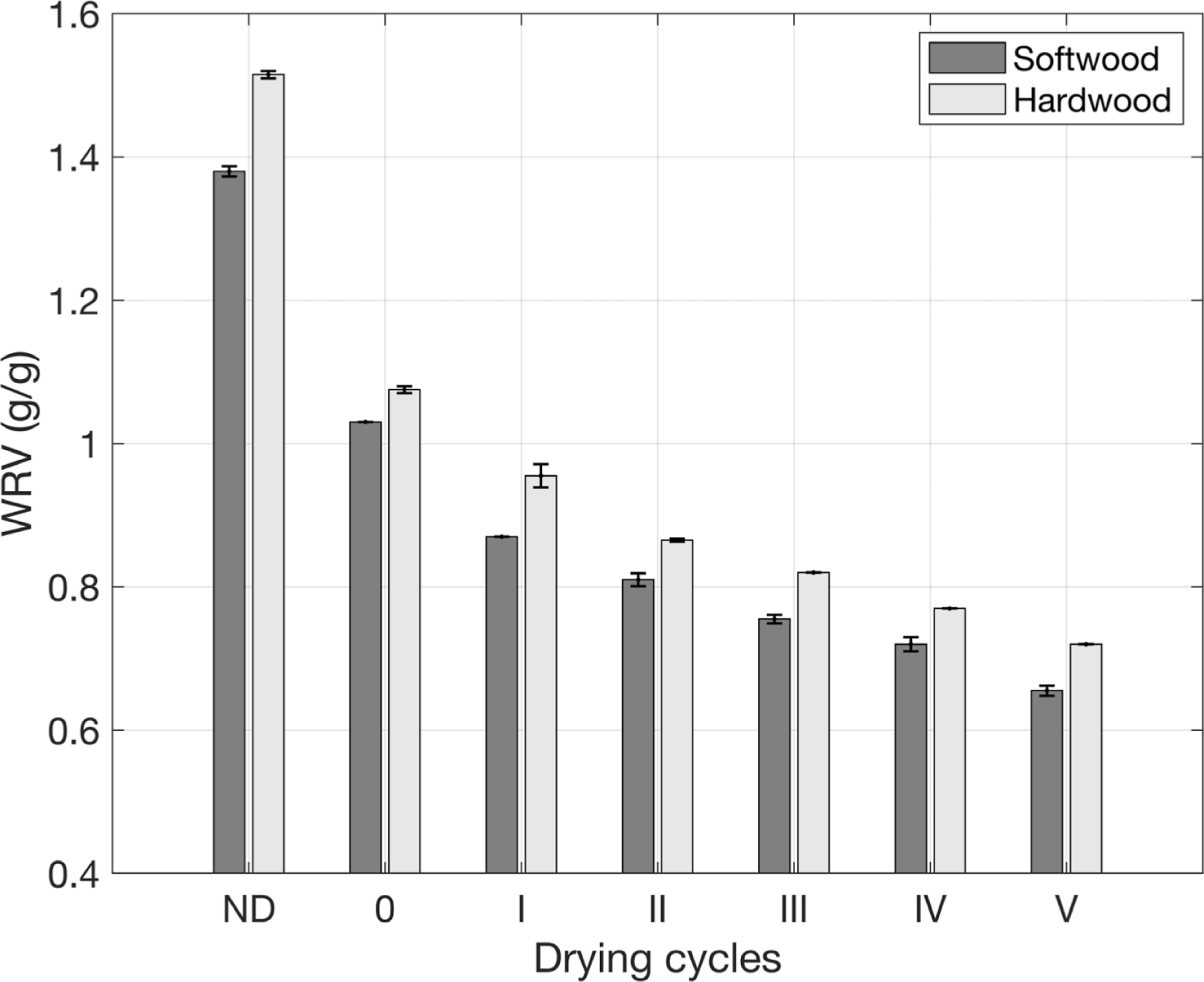
WRV for the drying cycles of the softwood and hardwood pulps; error bars represent the range of half the distance between max and min values.

When hornification is increased by successive drying cycles, the xyloglucan (XG) adsorption is decreased, as seen in Figure 2. The never-dried (ND) samples have the largest xyloglucan-accessible surfaces, followed by a large decrease in adsorbed XG observed for the first drying and later a linear decrease with each cycle. This suggests that most of the accessible surface area is lost after the first drying cycle. One explanation for this is that subsequent drying cycles reduce the size of the pores previously accessible to XG, but this reduction does not hinder the penetration of water molecules. In addition, it is clear that drying temperature has a significant effect on hornification and the ability to reswell and bind XG.

**Figure 2:**
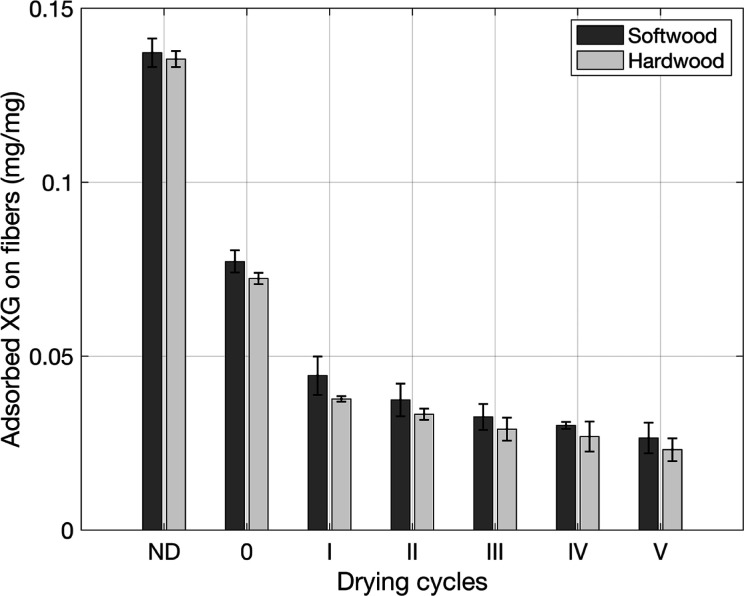
Xyloglucan adsorption for the drying cycles of the softwood and hardwood pulps, with error bars representing the standard deviation based on 4 samples.

Xyloglucan adsorption results are expressed as mg of XG adsorbed per mg of fiber or xyloglucan-accessible surface area. The surface accessible to the XG molecule is highly dependent on the concentration from which it is adsorbed; at lower concentrations, the xyloglucan molecule can adopt a more extended configuration on the cellulose surface, while at higher concentrations, the likelihood of loops and tails is more pronounced.^30^ Calculating an absolute value for the specific surface area is, therefore, not possible under these circumstances. In addition, the xyloglucan molecules are too large to allow the xyloglucan molecules to enter smaller pores. Therefore, xyloglucan-accessible areas should be considered as an estimate of free cellulose surface area but not as an absolute value.

The hornification ratio is a new metric introduced to quantify the effects of drying on the water retention capacity of fiber-based materials. It is defined as the ratio between the water retention value (WRV) of never-dried samples and that of dried samples. Mathematically it is expressed by eq 1.

1 $$Hornification\;Ratio=\frac{{WRV}_{never-dried}}{{WRV}_{dried}}$$

The hornification ratio provides a convenient and straightforward way of assessing the extent of irreversible changes associated with hornification. Unlike absolute WRV values, which can vary significantly depending on experimental conditions and material types, the hornification ratio normalizes the comparison by focusing on the relative change caused by drying. A lower hornification ratio indicates minimal loss of water retention capacity, suggesting that the fiber network retains much of its original porous structure and flexibility. Conversely, a higher ratio indicates significant structural collapse and a loss of hydrophilic sites due to drying. If the swelling behavior is unchanged and hornification is zero, the hornification ratio is equal to one, and for negative hornification,^17^ the hornification ratio is less than one.

By offering a simple yet robust measure of drying-induced changes, the hornification ratio enables a systematic evaluation of the effects of drying.

When calculating the linear regression for all values of HW and SW, WRV and xyloglucan adsorption show a close overall correlation of *R*_HW_^2^ = 0.97 and *R*_SW_^2^ = 0.98 for the range of samples tested in this study. This shows that the accessible cellulose surfaces and WRV are closely correlated in the case of hornification and supports the idea that hornification is a collapse of the fiber wall structure and thus a reduction in surface area. Xyloglucan adsorption provides a robust complementary characterization technique for monitoring hornification or surface area loss.

The split linear regressions in Figure 3 have been fitted to fit the shape of a two-slope linearity with a kink around 0.04 mg/mg adsorbed XG on fiber surfaces observed in the data, and the *R* ^2^ values are close to 1 for all four parts. Drying temperature has previously been shown to affect hornification mechanisms, becoming much more pronounced at temperatures higher than 100 °C.^16^ Plotting the same data as in Figure 3 with linear regressions split at 100 °C gives one potentially plausible representation of how the hornification ratios fit with the XG method (Figure 3). The differing slopes of the trend lines may reflect a temperature-dependent collapse of pores of varying sizes as higher temperatures could cause the closure of larger or more resilient pores. However, care should also be taken when reading these data as there is only a single data point at 20 °C, which in Figure 3 is placed in the same series as those at 125 °C. It is likely that drying cycles below and above 100 °C follow separate power-law relationships that do not overlap.

**Figure 3:**
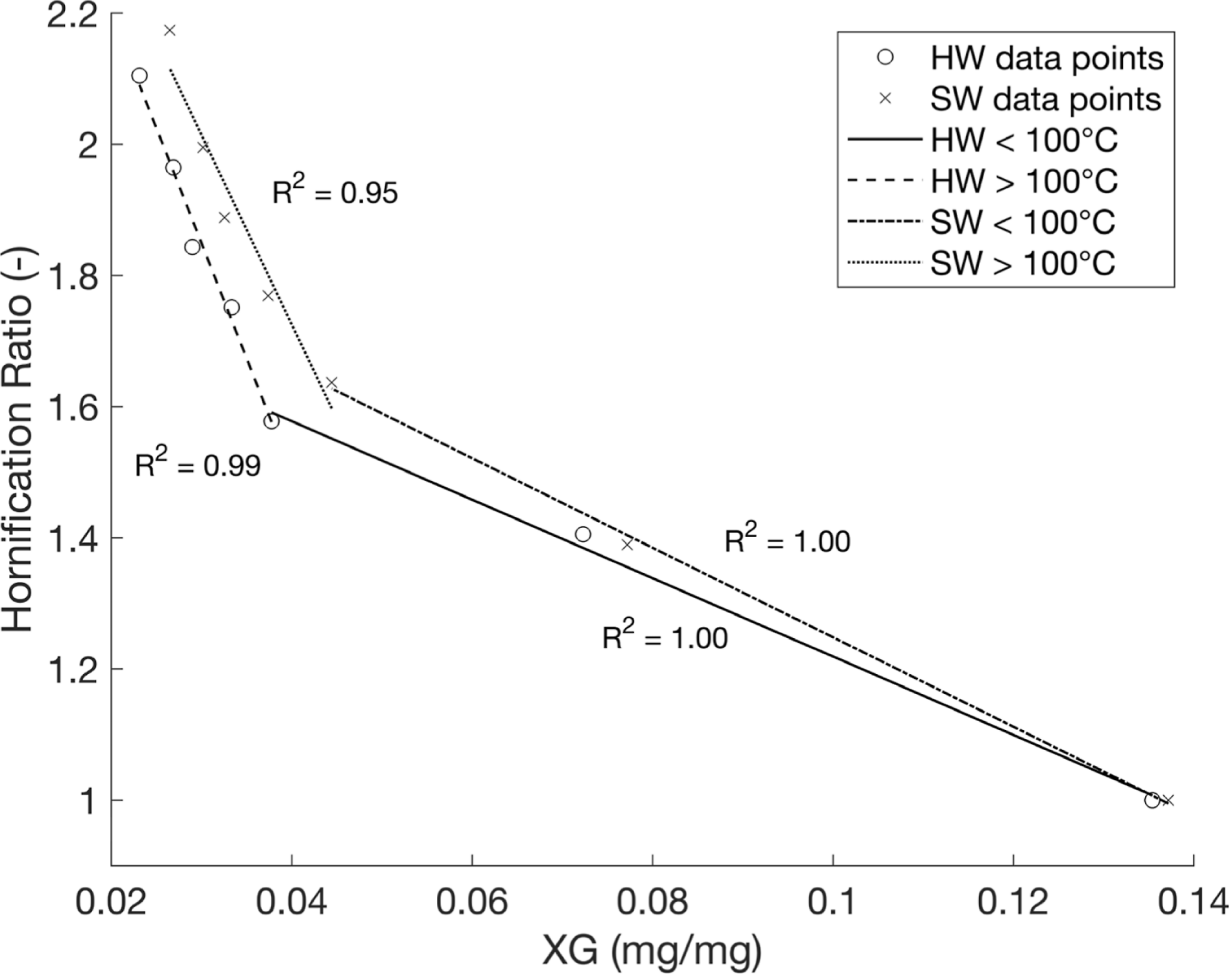
Hornification ratio and amount of xyloglucan adsorbed on fibers for all samples, linear regressions for each pulp, plotted at 100 °C. Note that the *R* ^2^ values for <100 °C are close to 1, although they are based on only 3 means, and it is clear from the figure that it is not a perfect fit.

Qualitative analysis using light microscopy remains inherently difficult to draw comprehensive conclusions about all the fibers in the sample. Nevertheless, subtle differences in the morphology of the limited number of fibers captured in the images were discernible. For a limited number of the most extreme drying differences in this study, never-dried (ND) fibers with five times high-temperature drying (V), the fibers in the images may appear slightly more damaged and crumpled after five consecutive drying cycles (Figure 4). These differences are very small indeed, and quantitative measurements were necessary as a complement to the images.

**Figure 4:**
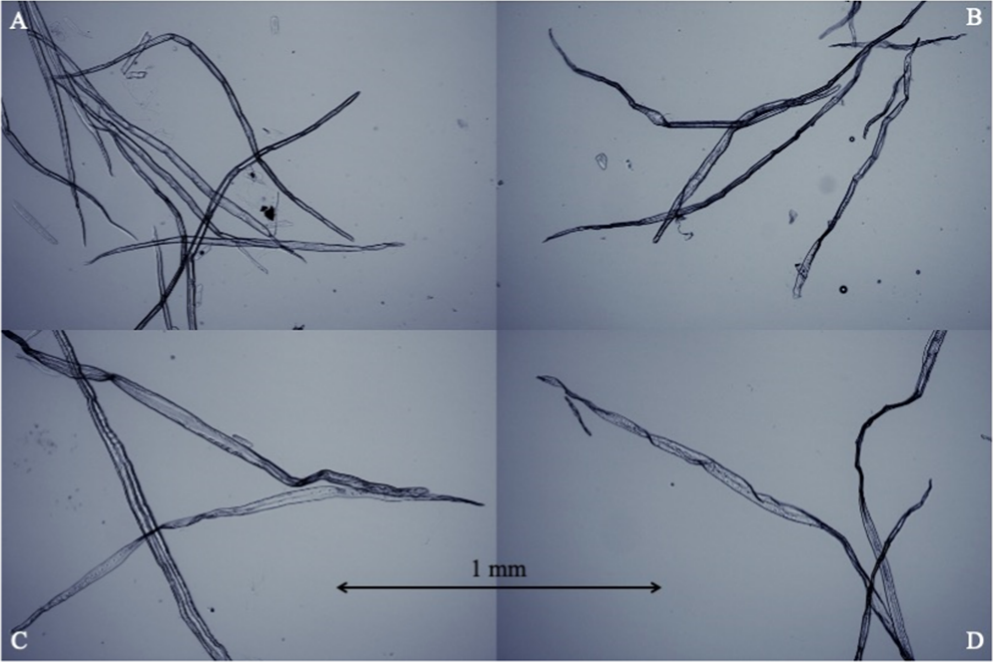
Optical microscopy images of never-dried hardwood fibers (A), five times high-temperature dried hardwood fibers (B), never-dried softwood fibers (C), and five times high-temperature dried softwood fibers (D).

To complement the qualitative measurements from the optical microscope, quantitative measurements were made with a Valmet FS5. The FS5 results for fiber length and width are shown in Figures 5–8, plotted against the WRV values. The results show a decrease in fiber width with drying cycles for the studied softwood pulp (Figure 8), which is consistent with previous studies.^14^ In addition, the hardwood pulp fibers in these experiments appear to shorten with drying cycles (Figure 5), which, to the authors’ knowledge, has not been reported previously. Fiber lengths for the softwood pulp and fiber widths for the hardwood pulp appear to be unaffected by sequential drying, based on the low correlation between the values (Figures 6 and 7).

**Figure 5:**
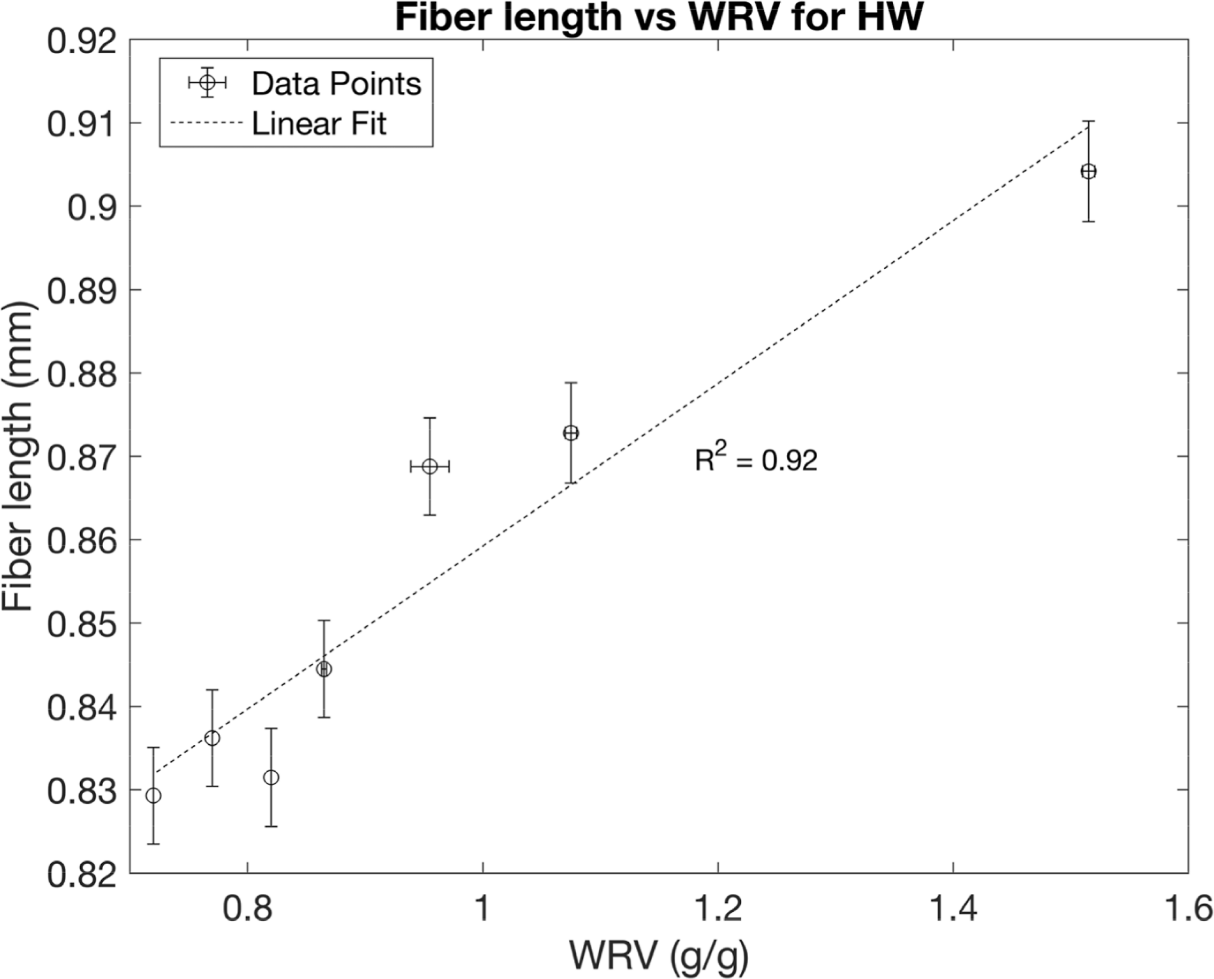
Fiber lengths plotted against WRV from different numbers of drying cycles for hardwood, with error bars for WRV representing the range of half the distance between the maximum and minimum values and error bars for fiber length representing a 95% confidence interval from 13000 observed particles.

**Figure 6:**
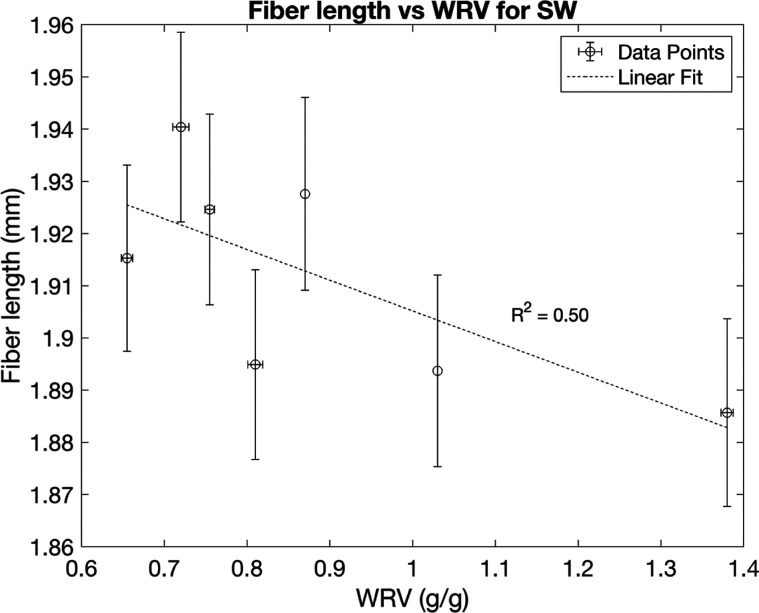
Fiber lengths plotted against WRV from different numbers of drying cycles for softwood pulps, with error bars for WRV representing the range of half the distance between the maximum and minimum values and error bars for fiber length representing a 95% confidence interval from 13000 observed particles.

**Figure 7:**
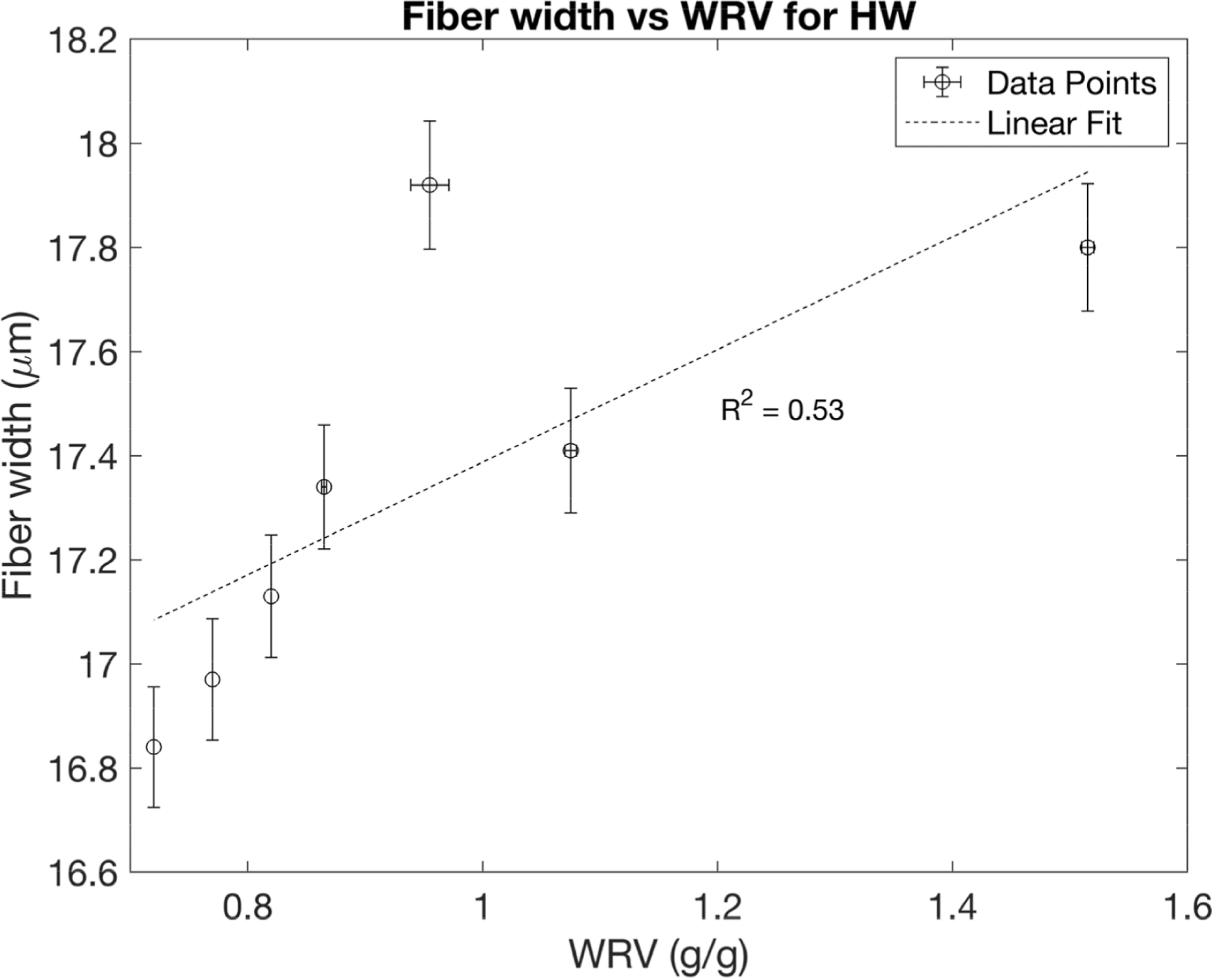
Fiber widths plotted against WRV from different numbers of drying cycles for hardwood pulps, with error bars for WRV representing the range of half the distance between the maximum and minimum values and error bars for fiber length representing a 95% confidence interval from 13000 observed particles.

**Figure 8:**
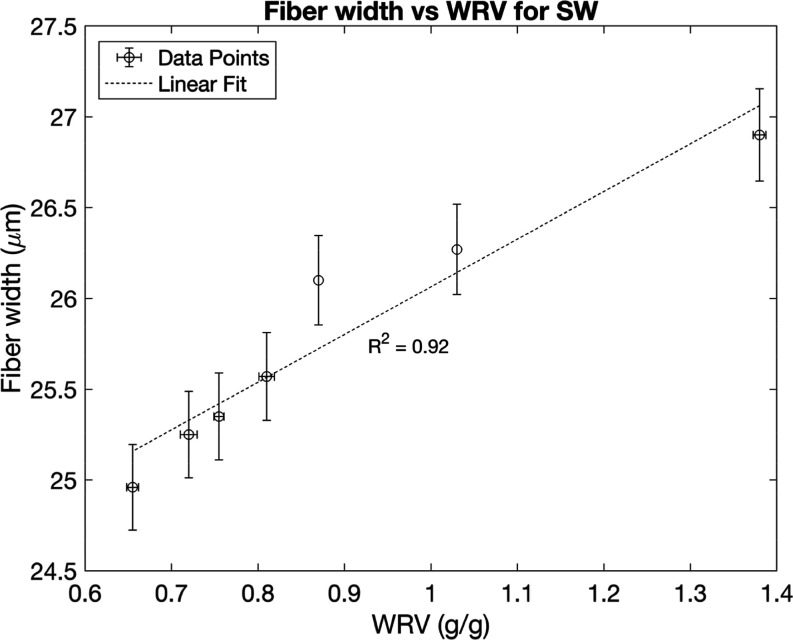
Fiber widths plotted against WRV from different numbers of drying cycles for softwood pulps, with error bars for WRV representing the range of half the distance between the maximum and minimum values and error bars for fiber length representing a 95% confidence interval from 13000 observed particles.

According to the theory of hornification,^1^ cellulose surfaces are drawn together during drying, and so it might be expected that dimensions would visibly decrease with increasing hornification. However, according to Weise and Paulapuro,^36^ hornification, defined as an irreversible reduction in the water retention value (WRV), is not directly related to shrinkage in the fiber width direction. Instead, other structural changes within the fibers contribute to hornification. This is observed in this study and is observed differently depending on the pulp type. Hardwood libriform fibers have relatively smaller lumens and therefore relatively thicker cell walls relative to fiber diameter than softwood tracheids.^37^ This may partially explain the observations: Thicker, more robust hardwood fibers with relatively more material in the transverse direction of the fibers (along the fiber width) may increase the possibilities for shrinkage. One explanation for this phenomenon is the difference in cellulose orientation and fibril angle between the softwood and hardwood pulp fibers. However, as no direct measurements of fibrillar angle were performed, we acknowledge that there is a need for further investigation into the role of fibrillar angle in fiber property development during drying. Additionally, fiber curl and its potential changes during drying could influence the measurement of fiber length. In particular, out-of-plane curling may introduce variations in the apparent fiber length as measured in two-dimensional imaging techniques. This aspect is relevant when interpreting fiber length measurements and could be explored in future studies to assess its significance in the context of drying-induced fiber modifications. Another theory is mechanical damage caused by the repeated cycles of drying and rewetting, where repeated disintegration damages the fibers and causes dimensional changes, although this does not explain the observed difference between hardwood and softwood.

Xylan is mainly found in hardwoods, where it plays a key role in influencing the arrangement of cellulose fibrils. This polysaccharide may promote a more even distribution of fibrils within the fiber structure, creating a more uniform and dispersed network. In contrast, glucomannan, which is abundant in softwoods, has the opposite effect, encouraging the cellulose fibrils to cluster together.^38^ This difference in chemical composition, including the fact that xylan is a charged polysaccharide, and the resulting fibril organization can have a significant impact on how fibers respond to environmental changes such as drying. For example, it could potentially explain differences in the way hardwood and softwood fibers shrink. Hardwood fibers should then be expected to have more evenly dispersed fibrils and therefore exhibit more uniform shrinkage, whereas more clustered fibrils in softwood fibers may lead to uneven dimensional changes. In particular, these structural differences may contribute to whether the fibers shrink primarily along their length or across their width,^38–42^ but further studies are needed to confirm or reject this hypothesis.

## Conclusions

Drying cycles increase hornification for both softwood and hardwood pulps; the greatest difference is for never-dried pulps and the first drying; then, reductions are linear for five high temperature drying cycles for both hardwood and softwood pulps.

Accessible cellulose surfaces are closely related to hornification, as shown by xyloglucan adsorption. The xyloglucan method correlates well with WRV and could therefore be used as a standalone method for measuring hornification.

The relationship between hornification ratios and accessible areas for xyloglucan adsorption shows slightly different linear relationships above and below 100 °C, which may indicate that the hypothesis of different hornification mechanisms at different temperatures shown in previous literature is correct, although more experimental evidence is needed to be certain.

Fiber width decreases with increasing hornification for the particular softwood pulp used in this study, and fiber length decreases with increasing hornification for the particular hardwood pulp. The dimensional differences of the fibers along with their decreasing water retention capabilities that come with increased hornification will give the pulps individually stronger and stiffer fibers but at the cost of fewer capabilities to form fiber–fiber bonds, resulting in mechanically weaker products of less density.
